# N-3 PUFA Supplementation Triggers PPAR-α Activation and PPAR-α/NF-κB Interaction: Anti-Inflammatory Implications in Liver Ischemia-Reperfusion Injury

**DOI:** 10.1371/journal.pone.0028502

**Published:** 2011-12-08

**Authors:** Jessica Zúñiga, Milena Cancino, Fernando Medina, Patricia Varela, Romina Vargas, Gladys Tapia, Luis A. Videla, Virginia Fernández

**Affiliations:** Molecular and Clinical Pharmacology Program, Institute of Biomedical Sciences, Faculty of Medicine, University of Chile, Santiago, Chile; Sun Yat-sen University Medical School, China

## Abstract

Dietary supplementation with the n-3 polyunsaturated fatty acids (n-3 PUFA) eicosapentaenoic acid (EPA) and docosahexaenoic acid (DHA) to rats preconditions the liver against ischemia-reperfusion (IR) injury, with reduction of the enhanced nuclear factor-κB (NF-κB) functionality occurring in the early phase of IR injury, and recovery of IR-induced pro-inflammatory cytokine response. The aim of the present study was to test the hypothesis that liver preconditioning by n-3 PUFA is exerted through peroxisone proliferator-activated receptor α (PPAR-α) activation and interference with NF-κB activation. For this purpose we evaluated the formation of PPAR-α/NF-κBp65 complexes in relation to changes in PPAR-α activation, IκB-α phosphorylation and serum levels and expression of interleukin (IL)-1β and tumor necrosis factor (TNF)-α in a model of hepatic IR-injury (1 h of ischemia and 20 h of reperfusion) or sham laparotomy (controls) in male Sprague Dawley rats. Animals were previously supplemented for 7 days with encapsulated fish oil (General Nutrition Corp., Pittsburg, PA) or isovolumetric amounts of saline (controls). Normalization of IR-altered parameters of liver injury (serum transaminases and liver morphology) was achieved by dietary n-3 PUFA supplementation. EPA and DHA suppression of the early IR-induced NF-κB activation was paralleled by generation of PPAR-α/NF-κBp65 complexes, in concomitance with normalization of the IR-induced IκB-α phosphorylation. PPAR-α activation by n-3 PUFA was evidenced by enhancement in the expression of the PPAR-α-regulated Acyl-CoA oxidase (Acox) and Carnitine-Palmitoyl-CoA transferase I (CPT-I) genes. Consistent with these findings, normalization of IR-induced expression and serum levels of NF-κB-controlled cytokines IL-lβ and TNF-α was observed at 20 h of reperfusion. Taken together, these findings point to an antagonistic effect of PPAR-α on NF-κB-controlled transcription of pro-inflammatory mediators. This effect is associated with the formation of PPAR-α/NF-κBp65 complexes and enhanced cytosolic IκB-α stability, as major preconditioning mechanisms induced by n-3 PUFA supplementation against IR liver injury.

## Introduction

Human liver resections involving vascular occlusion to reduce blood loss may lead to severe hepatic dysfunction, with irreversible organ damage due to hepatocyte and endothelial cell death [1]. Taking into account that vascular occlusion of the liver or ischemia (I), followed by its restoration during reperfusion (R) occurs during surgical manoeuvres such as transplantation, tissue resection under inflow occlusion (Pringle manoeuvre), and hypoperfusion shock, several preconditioning strategies affording resistance to liver IR injury have been evaluated [2]. In this respect, we have established that dietary supplementation with the n-3 polyunsaturated fatty acids (n-3 PUFA) eicosapentaenoic acid (EPA) and docosahexaenoic acid (DHA), which are highly concentrated in fish oils, affords significant prevention of liver injury induced by IR in the rat, thus representing a novel preconditioning strategy [3]. Fish oil supplementation significantly enhanced the hepatic content of n-3 PUFAs, with diminution in the n-6/n-3 PUFA ratio, suppression of IR-induced oxidative stress, and recovery of IR-altered pro-inflammatory cytokine response and nuclear factor-κB (NF-κB) functionality [3]. In the latter case, n-3 PUFA supplementation normalized both the early increase (3 h) and late diminution (20 h) in NF-κB DNA activity induced by IR [3].

As a result of their incorporation into cell phospholipids, EPA and DHA exert a significant inhibition of the metabolism of the n-6 PUFA arachidonic acid (AA), thus decreasing the release of AA-derived pro-inflammatory eicosanoids [4]. In addition, EPA and DHA have been shown to generate a group of lipid mediators called resolvins (E- and D-series) and protectins with potent anti-inflammatory and inflammation resolution properties [4], [5]. Studies in experimental models of liver injury have reported beneficial actions of n-3 PUFA-derived resolvins and protectins, preventing liver DNA damage and oxidative stress, with significant reduction in necroinflammatory liver injury and hepatic steatosis [6], [7]. Although these mediators might explain many of the anti-inflammatory actions of n-3 fatty acids, eicosanoid-independent actions including EPA and DHA effects on transcription factors regulating inflammatory gene expression such as NF-κB, should be considered. Supporting this view are the data showing the decreasing effect of n-3 PUFAs on the production of pro-inflammatory cytokines regulated by NF-κB [8].

NF-κB is an essential factor with dual intracellular effects, playing a role in acute cellular stress responses by inducing proteins affording survival [9], or acting as a pro-inflammatory transcription factor by upregulating the expression of pro-inflammatory cytokines and adhesion molecules [10]. Changes in NF-κB DNA binding activity are main mediators in liver IR injury, as pointed by its biphasic activation pattern in liver IR injury in the rat. An early peak (0.5–3 h after reperfusion) due to the nuclear translocation of NF-κB p50/p65 heterodimers correlates with the acute phase of IR injury, is followed by a second peak (9–12 h after reperfusion) [10] and a significant diminution thereafter (18–20 h after reperfusion) [11].

On the other hand, peroxisome proliferator-activated receptor α (PPAR-α), a ligand-activated transcription factor highly expressed in the liver, is activated by several PUFAs [12]. EPA and DHA are considered as PPAR-α agonists and inhibitors of NF-κB DNA binding activity [8]. PPAR-α agonists, such as fibrates, reduce inflammatory responses at the vascular, splenic, and hepatic level by down-regulation of cytokine-induced genes, an effect that was attributed to PPAR-α direct protein-protein interaction with NF-κB subunit p65, thus diminishing NF-κB DNA binding [13]. In addition to the antagonistic action on NF-κB signalling, PPAR-α activators are known to induce inhibitor of NF-κB, IκB-α, in primary smooth muscle cells and hepatocytes, which is associated with reduced NF-κB DNA-binding triggered by PPAR-α [14]. Furthermore, inhibition of both IκB kinase (IKK) activity and TNF-α-induced IκB-α phosphorylation by fenofibrate in human umbilical vein endothelial cells has been reported, an effect leading to enhanced cytosolic IκB-α stability and promotion of NF-κB cytoplasmic sequestering through its tight association with IκB-α [15]. Similarly to the effects of fibrates, n-3 PUFAs can also decrease the expression of pro-inflammatory genes, as shown by the decreased TNF-α expression by EPA preventing IκB phosphorylation and NF-κB translocation into the nucleus [8].

Considering PPAR-α anti-inflammatory effect in relation to decreased NF-κB DNA binding capacity, in the current investigation we tested the hypothesis that liver preconditioning by dietary n-3 PUFAs is exerted through PPAR-α activation and interference with that of NF-κB. For this purpose, we evaluated PPAR-α activity by the expression of PPAR-α-regulated proteins in an experimental model of n-3 PUFA preconditioning against liver IR injury. We also explored the antagonic action of PPAR-α on NF-κB signalling pathway by assessing the formation of PPARα/NF-κBp65 complexes and changes in IκB-α phosphorylation, in relation to changes in the expression of pro-inflammatory cytokines regulated by NF-κB. We revealed that PPAR-α/NF-κBp65 complex generation and enhanced cytosolic IκB-stability mediated the antagonistic effects of PPAR-α on NF-κB-controlled transcription of pro-inflammatory cytokines, thus constituting major preconditioning mechanisms against IR liver injury induced by n-3 PUFA supplementation.

## Materials and Methods

### Animal preparation

Weaning male Sprague-Dawley rats (Bioterio Central, ICBM, Faculty of Medicine, University of Chile) were allowed free access to a specially formulated diet (20% casein, 10% n-6 PUFAs, lipo/hydrosoluble vitamins and minerals, Department of Nutrition, Faculty of Medicine, University of Chile). Animals received water *ad libitum* and were housed on a 12-h light/dark cycle. At day 15, the n-3 PUFA groups were supplemented for 7 days with encapsulated fish oil (General Nutrition Corp., Pittsburg, PA) and the control groups received isovolumetric amounts of saline, thus comprising four experimental groups: (a) Control-Sham, (b) Control-IR, (c) (EPA+DHA)-Sham and (d) (EPA+DHA)-IR. In these conditions the n-3 PUFA groups received EPA (270 mg/kg) and DHA (180 mg/kg).

### Model of partial ischemia-reperfusion injury

At day 8 after EPA plus DHA supplementation, rats were anaesthetized with intraperitoneal (1 ml/kg) zolazepam chlorhydrate (25 mg/ml) and tiletamine chlorhydrate (25 mg/ml) (Zoletil 50; Virbac S/A, Carros, France) and IR was induced by temporarily occluding the blood supply to the left and median lobes of the liver by means of a Schwartz clip (Fine Science Tools, Vancouver, BC, Canada) for 1 h, followed by up to 20 h of reperfusion, as previously described [11]. Control animals were subjected to anaesthesia and sham laparotomy. To evaluate liver preconditioning by dietary n-3 PUFAs, blood samples for serum AST and ALT (specific diagnostic kits; Biomerieux SA, Marcy l' Etoile, France) and the NF-κB dependent cytokines TNF-α and IL-1β (Biosource International, Camarillo, CA, USA) assessment were obtained by cardiac puncture at 20 h of reperfusion. Liver samples were obtained at 3 h of reperfusion, for assessments of NF-κB DNA binding, PPAR-α/NF-κBp65 complexes, changes in IκBα phosphorylation, and expression of PPAR-α regulated proteins (Acyl-CoA oxidase [Acox] and Carnitine-Palmitoyl-CoA transferase I [CPT-I]). Liver samples obtained at 20 h of reperfusion were used for assessments of TNF-α and IL-1β expression and liver morphology. Liver samples were taken from the medial lobes, and either frozen in liquid nitrogen and stored at −80°C, for cytokines assays or fixed in phosphate-buffered formalin, embedded in paraffin, and stained with hematoxylin-eosin for morphological assessments.

### Ethics Statement

Experimental animal protocols and animal procedures complied with the Guide for the Care and Use of Laboratory Animals (National Academy of Sciences, NIH Publication 6–23, revised 1985) and were approved by the “Bioethics Committee for Research in Animals”, Faculty of Medicine, University of Chile (CBA 0381 FMUCH).

### Assessments of NF-κB DNA binding and PPAR-α/NF-κBp65 complexes

For these studies nuclear protein extracts from liver samples obtained at 3 hours of reperfusion, were prepared according to Deryckere and Gannon [16]. NF-κB DNA binding was determined by electromobility shift assay, using the NF-κB probe 5′-GAT CTC AGA GGG GAC TTT CCG AG-3′ (Invitrogen Life Technologies, Carlsbad, CA), labelled with α-^32^PdCTP and the Klenow DNA Polymerase Fragment I (Invitrogen Corp., Carlsbad, CA), as described previously [17]. The specificity of the reaction was determined by a competition assay using 100-fold molar excess of unlabelled DNA probe. The sub-unit composition of DNA binding protein was confirmed by supershift assay using specific antibodies from goat and rabbit IgG raised against NF-κB p50 and p65 (Santa Cruz Biotechnology, Santa Cruz, CA). Samples were loaded on non-denaturating 6% polyacrylamide gels and run until the free probe reached the end of the gel; NF-κB bands were detected by autoradiography and quantified by densitometry using Scion Image (Scion Corp., Frederick, MD). PPAR-α/NF-κBp65 complexes were evaluated by co-immunoprecipitation. For this purpose 350 µg of nuclear protein extracts [16] were homogenized in lysis buffer (1%NP-40, 0.15 mol/l NaCl, 0.01 mol/L NaH_2_PO_4_ pH 7.2, 0.2 milimoles/L EDTA, 0.5 milimoles/L phenylmethylsulfonyl fluoride or PMSF and 5 µg/ml pesptatine, leupeptine and aprotinine), incubated at 0°C and clarified by centrifuging (13,000 g; 30 min). Twenty µg of rabbit polyclonal antibodies, raised against NF-κB or PPAR-α (Abcam, Cambrige, UK), were precleared and linked to sepharose-protein A beads (GE, Amersham Biosciences, Uppsala, Sweden), by incubation and rotation for 2 h at room temperature. Nuclear lysates were separately incubated (4°C, overnight, with end-over-end rotation) with NF-κB or PPAR-α precleared antibodies. The immune complexes were collected by centrifugation at 1,000 g for 1 min in an Eppendorf refrigerated centrifuge, rinsed twice (200 µl phosphate buffer), resuspended in Laemmli sample buffer (100°C for 5 min), separated in 10% polyacrylamide gels using SDS-PAGE [18] and immunoblotted on nitrocellulose membranes (input corresponded to 10% of initial extract) [19]. The membranes were hybridized with NF-κB and PPARα antibodies (both antibodies for each membrane). The proteins were visualized by enhanced chemiluminescent detection (Pierce Biotechnology).

### Western blot analysis of IκB-α phosphorylation

Liver samples (100–500 mg) frozen in liquid nitrogen were homogenized and suspended in a buffer solution pH 7.9, containing 10 mM Hepes, 1 mM EDTA, 0.6% NP-40, 150 mM NaCl, and 0.5 mM PMSF, followed by centrifugation (3,020 g for 5 min). Soluble protein fractions (50 µg) were separated on 12% polyacrylamide gels using SDS–PAGE (18) and transferred to nitrocellulose membranes [19] which were blocked for 1 h at room temperature with TBS containing 4.5% bovine serum albumin. The blots were washed with TBS containing 0.1% Tween 20, hybridized with rabbit polyclonal primary antibodies, for either non-phosphorylated IκB-α (IκBα-OH) or phosphorylated-IκB-α (IκBα-OP) (Dako Corp., Carpinteria, CA, USA) and incubated (overnight at 4°C for IκBα-OH and 76 h at 4°C for IκBα-OP). After extensive washing, the antigen–antibody complexes were detected using horseradish peroxidase-labelled goat anti-rabbit IgG or goat anti-mouse IgG and a SuperSignal West Pico chemiluminescence kit detection system (Pierce, Rockford, IL, USA). In all determinations, mouse monoclonal antibody for rat β-actin (ICN Biomedicals, Inc., Aurora, OH) was used as internal control.

### RT-PCR assay of Acox, CPT-I, TNF-α and IL-1β mRNA expression

PPAR-α regulated proteins (Acox and CPT-I) and NF-κB regulated cytokines (TNF-α and IL-lβ) were assessed by RT-PCR assay. Total RNA was isolated from 15–25 mg of frozen liver using an RNAqueous®-4PCR Kit (Ambion, Inc., Austin, Tx, USA) according to the manufacturer's instructions. Quantification of total RNA was performed spectrophotometrically (A260/A280 ratio) and RNA quality was checked by electrophoresis on 0.8% agarose gels, using a molecular size marker. The resulting DNAse-free RNA was reverse-transcribed to cDNA with Superscript II reverse transcriptase (Invitrogen Corp., Carlsbad, CA, USA), according to the manufacturer's instructions, and random hexamer primers (pd[N]6) (Promega, Madison, WI, USA). The resulting cDNA was amplified in a PCR reaction using Platinum® Taq (Invitrogen Corp., Carlsbad, CA, USA), according to the manufacturer's instructions, and control 18S Classic II (QuantumRNA™ Classic 18S). Nucleotide sequences for sense and antisense primers used in this study were 5′-GAG CCA CGA AGC CCT CAA AC-3′ and 5′-GTG GCC TCA CAG ATT CCA GG-3′ for CPT-I; 5′-GTT GAT CAC GCA CCAT CTT GG-3′ and 5′-GCG TGA TTG GAA GTT TTC CC-3′ for Acox; 5′-ACT GAA CTT CGG GGT GAT CG-3′ and 5′-TAC ATG GGC TCA TAC CAG GG C-3′ for TNF-α; 5′-TTC TTT GAG GCT GAC AGA CC-3′ and 5′-CGT CTT TCA TCA CAC AGG AC-3′ for IL-1β. For amplification a thermocycler T personal, Biometra® was used. The amplification was initiated by 5 minutes of denaturation (94°C), followed by 32 cycles (94°C for 4 min, 37°C for 30 s, 59°C for 30 s, 72°C for 1 min, 72°C for 10 min) for CPT I; 30 cycles (94°C for 4 min, 37°C for 30 s, 55°C for 30 s,72°C for 1 min, 72°C for 10 min) for Acox; 40 cycles (94°C for 4 min, 37°C for 30 s, 57°C for 30 s,72°C for 1 min, 72°C for 10 min) for TNF-α; 44 cycles (94°C for 4 min, 37°C for 30 s, 55°C for 30 s,72°C for 1 min, 72°C for 10 min) for IL-1β. All amplification products were stored at 4°C before the electrophoretic step. All PCR products were electrophoresed on 1.2% agarose gels containing ethidium bromide, visualized by UV-induced fluorescence, and analyzed by densitometry using Scion Image (Scion Corp., Frederick, MD, USA).

### Statistical analyses

Values shown represent the mean ± SEM for the number of separate experiments indicated. Student's *t*-test for unpaired data or one-way ANOVA (GraphPad Prism 4.0 software, GraphPad Software, Inc. San Diego, USA) and the Newman-Keuls test assessed the statistical significance of differences between mean values, as required. A *p*-value of less than 0.05 was considered significant.

## Results

**Figure 1:**
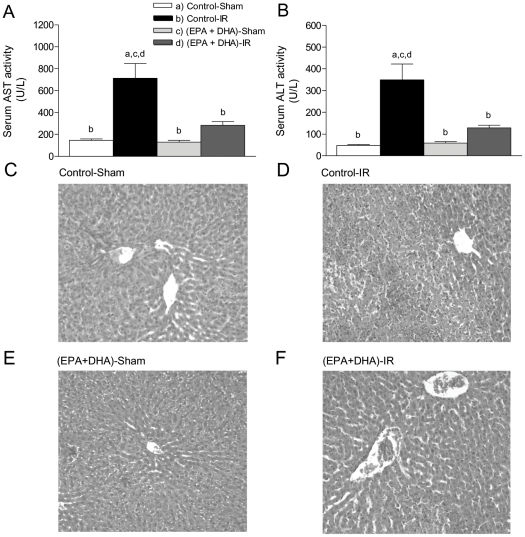
Effect of EPA plus DHA supplementation on serum (A) AST and (B) ALT levels, and (C–F) liver histologies after hepatic ischemia (1 h) – reperfusion (20 h) (IR). Values of serum transaminases (A and B) correspond to the means ± SEM for 9 to 18 rats per experimental group, and significance assessed by one-way ANOVA and the Newman-Keuls, 534 test (*p*<0.05) is shown by the letters identifying each experimental group. Representative liver sections from a control-sham rat (C), a control-IR animal (D), a (EPA plus DHA)-sham rat (E) and a (EPA plus DHA)-IR animal (F) (haematoxylin-eosin liver sections from a total of five animals per experimental group; original magnification ×100).

**Figure 2:**
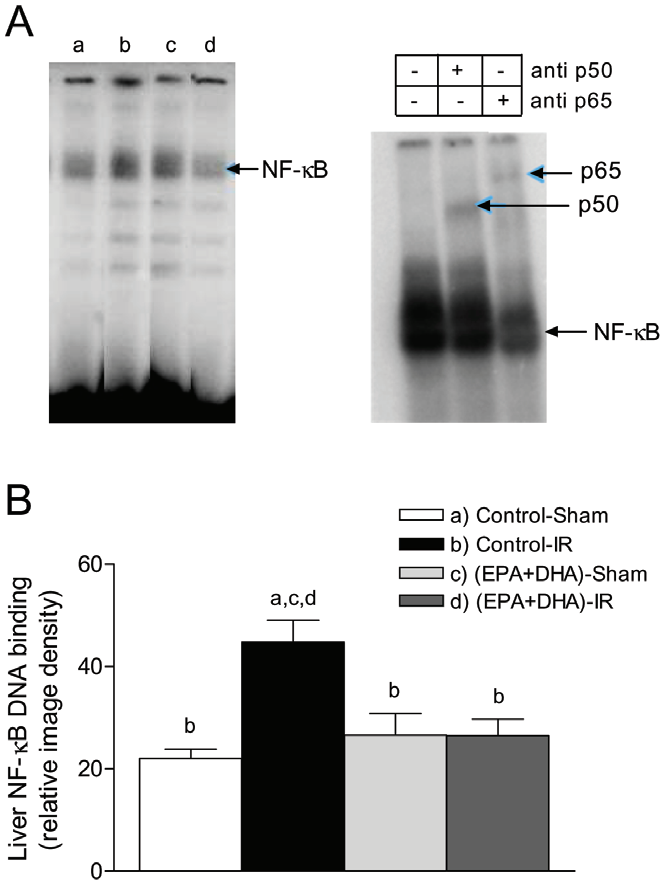
Effect of EPA plus DHA supplementation on liver NF-κB DNA binding after hepatic ischemia (1 h) - reperfusion (3 h) (IR). (A) Autoradiographs representing lanes loaded with 8 µg nuclear protein from an animal of each experimental group, and supershift analysis of a sample from a control-IR rat incubated with the labeled probe for NF-κB and with antibodies specific for NF-κB p50 (anti p50) and NF-κB p65 (anti p65). (B) Bar graphs corresponding to densitometric quantification of relative NF-κB DNA binding. Values shown correspond to the means ± SEM for 6 to 8 rats per experimental group, and significance assessed by one-way ANOVA and the Newman-Keuls, test (*p*<0.05) is shown by the letters identifying each experimental group.

In agreement with our previous reports [3] EPA plus DHA supplementation led to serum AST (Figure 1A) and ALT (Figure 1B) values comparable to those in control-sham-operated animals. Control rats subjected to IR exhibited a 4.5- and 7.3-fold increases (*p*<0.05) in serum AST and ALT at 20 h of reperfusion in relation to control-sham-operated animals, and effect that was suppressed by n-3 PUFA supplementation (Figures 1A and 1B, respectively). In agreement with these data, liver histological assessments showed normal liver morphology in control-sham and EPA plus DHA-sham groups (Figures 2C and 2E, respectively), whereas substantial distortion of liver architecture, degenerative changes with extensive areas of hepatocyte necrosis and apoptosis was observed in non-supplemented animals (control-IR group, Figure 2D). On the contrary, the livers of the EPA plus DHA-IR group showed normal architecture, with minimal-to moderate necrosis (Figure 2 F).

**Figure 3:**
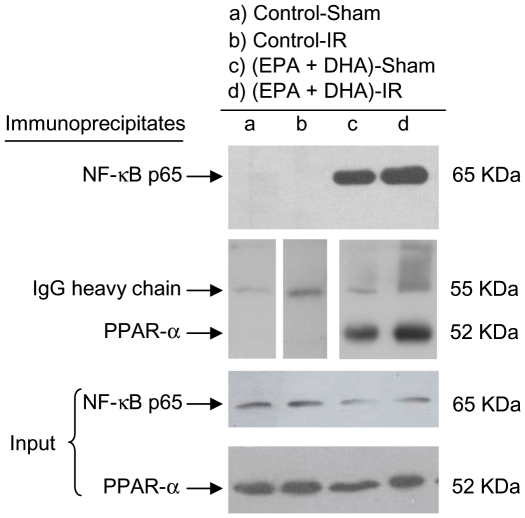
Effect of EPA plus DHA supplementation on the interaction of liver NF-κBp65 and PPARα (PPARα/NF-κBp65 complexes) after hepatic ischemia (1 h) - reperfusion (3 h) (IR). Nuclear protein extracts were subjected to immunoprecipitation with anti-PPARα or anti-NF-κBp65, linked to sepharose-protein A beads. Panel represents westernblot analysis of anti-PPARα-PPARα/NF-κBp65, anti-NF-κBp65-NF-κBp65/PPARα immune complexes, hybridized with NF-κB and PPARα antibodies, respectively, and the input (10% of the initial extract). 55 KDa band corresponds to IgG heavy chain.

**Figure 4:**
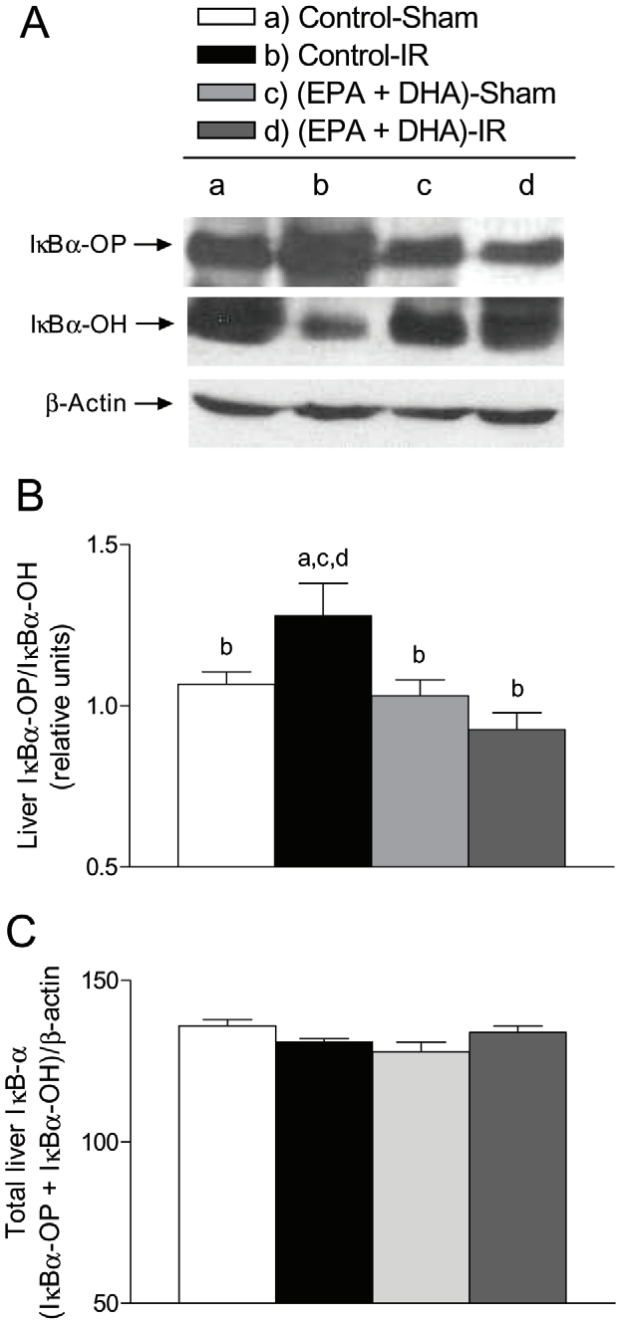
Effect of EPA plus DHA supplementation on liver IκBα phosphorylation after hepatic ischemia (1 h) - reperfusion (3 h) (IR). A. Liver contents of IκBα phosphorylated (IκBα-OP) and non phosphorylated (IκBα-OH) forms evaluated by Western blotting after hepatic ischemia-reperfusion (IR) injury in unpreconditioned and EPA plus DHA preconditioned rats. Representative blots of IκBα-OP, IκBα-OH and β-actin protein expression are shown, using 50 µg of soluble protein from a different rat of each group studied. B. Bar graphs correspond to the respective densitometric quantification expressed as IκBα-OP/IκBα-OH ratio expressed as means ± SEM for 6 different animals. C. Bar graphs correspond to the respective densitometric quantification expressed as (IκBα-OP+IκBα-OH)/β-actine ratios, expressed as means ± SEM for 6 different animals; significance studies (*p*<0.05; one-way ANOVA and the Newman-Keuls' test) are indicated by the letters identifying each experimental group.

**Figure 5:**
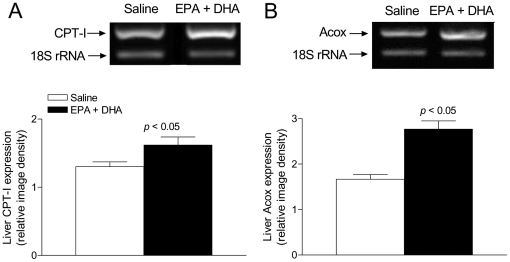
Effect of EPA plus DHA supplementation on liver Acyl-CoA oxidase (Acox) and Carnitine-Palmitoyl-CoA transferase I (CPT-I) mRNA expression. (A) Representative agarose gel electrophoresis for the RT-PCR products for CPT-I mRNA (509 bp) and for 18S rRNA (324 bp) after ethidium bromide staining in total hepatic RNA samples from control rats and EPA plus DHA preconditioned animals and densitometric quantification of RT-PCR products of the mRNA of CPT-I expressed as CPT-I mRNA/18S rRNA ratios to compare lane–lane equivalents in total RNA content. B. Representative agarose gel electrophoresis for the RT-PCR products for Acox mRNA (510 bp) and for 18S rRNA (324 bp) after ethidium bromide staining in total hepatic RNA samples from control rats and EPA plus DHA preconditioned animals and densitometric quantification of RT-PCR products of the mRNA of Acox expressed as Acox mRNA/18S rRNA ratios to compare lane–lane equivalents in total RNA content. Each data point represents the mean ± SEM for 3–8 different animals. Significance studies: *p<0.05 versus controls by Student's *t*-test for unpaired data.

**Figure 6:**
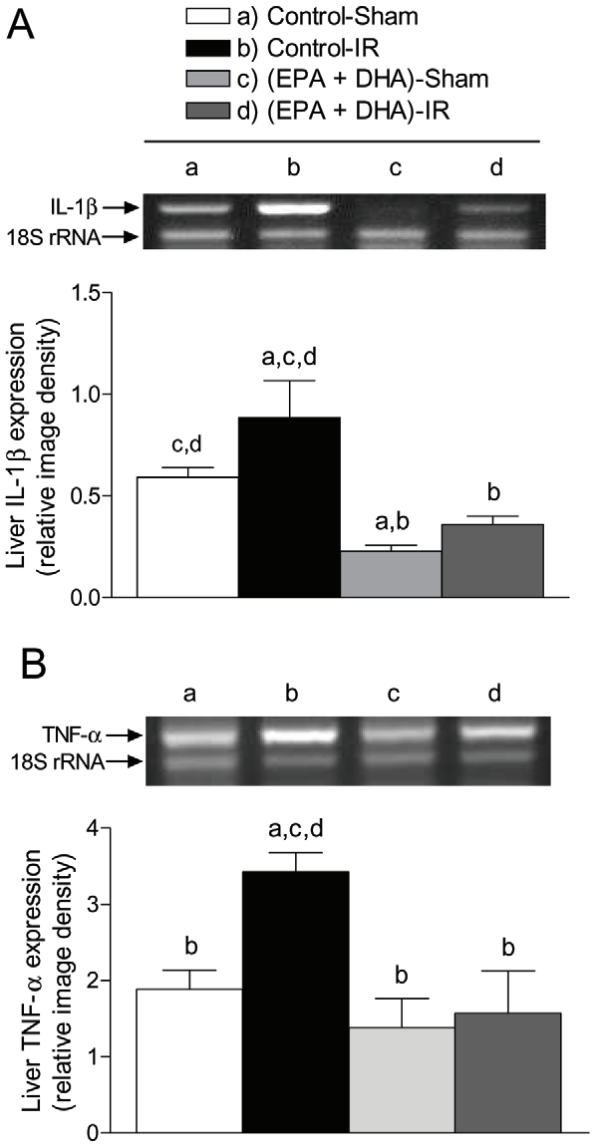
Effect of EPA plus DHA supplementation on liver interleukine (IL)-lβ and tumor necrosis factor (TNF)-α mRNA expression after hepatic ischemia (1 h) - reperfusion (20 h) (IR). (A) Representative agarose gel electrophoresis for the RT-PCR products for IL-lβ mRNA (511 bp) and for 18S rRNA (324 bp) after ethidium bromide staining in total hepatic RNA samples from control rats and EPA plus DHA preconditioned animals and densitometric quantification of RT-PCR products of the mRNA of IL-lβ expressed as IL-lβ mRNA/18S rRNA ratios to compare lane–lane equivalents in total RNA content. B. Representative agarose gel electrophoresis for the RT-PCR products for TNF-α mRNA (441 bp) and for 18S rRNA (324 bp) after ethidium bromide staining in total hepatic RNA samples from control rats and EPA plus DHA preconditioned animals and densitometric quantification of RT-PCR products of the mRNA of TNF-α expressed as TNF-α mRNA/18S rRNA ratios to compare lane–lane equivalents in total RNA content. Each data point represents the mean ± SEM for 3–9 different animals. ^a^ *p*<0.05 versus control sham-operated rats. ^b^ *p*<0.05 versus control animals subjected to IR. ^c^ *p*<0.05 versus [EPA plus DHA]-Sham -operated rats. ^d^ *p*<0.05 versus [EPA plus DHA] treated animals subjected to IR.

At 3 h of reperfusion, NF-κB DNA binding activity in control rats subjected to IR was increased by 100% (*p*<0.05) in relation to that in sham-operated controls, an effect that was suppressed by EPA plus DHA supplementation, without significant changes in sham-operated animals (Figure 2A and 2B), thus confirming our previous studies [3]. EMSA evaluation of NF-κB DNA binding activity included supershift analysis which confirmed the presence of NF-κB p50 and p65 (Figure 2A). Also, at 3 h of reperfusion direct interaction of PPAR-α and NF-κBp65 was detected in all liver samples from EPA plus DHA supplemented animals ([EPA plus DHA]-Sham and [EPA+DHA]-IR), leading to the formation of PPAR-α/NF-κBp65 complexes (Figure 3). PPAR-α/NF-κBp65 complexes were not detected in animals without EPA plus DHA supplementation (Figure 3) and western blot bands of NF-κBp65 of less intensity were detected in EPA plus DHA supplemented animals (Figure 3, input lower panel). In addition, significant 34% increase (*p*<0.05) in IκB-α phosphorylation, expressed as IκBα-OP/IκBα-OH ratios was observed in control animals subjected to IR over that in sham-operated controls, an effect that was suppressed by EPA plus DHA supplementation (Figure 4A and 4B). No changes in the total content of IκB-α (IκBα-OP+IκBα-OH) were observed among the experimental groups (Figure 4C). Considering that these results suggest antagonic actions of PPAR-α on NF-κB activation at 3 h of reperfusion in n-3 PUFA supplemented animals as a result of PPARα activation triggered by n-3 PUFA, changes in liver expression of PPAR-α-regulated CPT-I and Acox were assessed. Both CPT-I and Acox mRNA expression assessed by RT-PCR were significantly increased by 24% and 66%, respectively (*p*<0.05) in the liver of n-3 PUFA supplemented animals compared to saline-controls (Figure 5A and 5B). We next assessed the antagonic effects of PPAR-α on NF-κB signalling pathway. For this purpose, changes in liver expression of the NF-κB-regulated cytokines IL-lβ and TNF-α were evaluated in liver samples at 20 h of reperfusion. IL-lβ (Figure 6A) and TNF-α (Figure 6B) mRNA expression assessed by RT-PCR were significantly enhanced in control rats subjected to IR by 49% and 82%, respectively (*p*<0.05), compared to the saline-sham group, an effect that was suppressed by EPA plus DHA supplementation, without significant changes in sham-operated animals (Figure 6A and 6B). Serum levels of these NF-κB dependent cytokines were also examined in this study. TNF-α (saline-sham, 32.84±0.28 [n = 6] pg/mL; saline-IR, 63.81±8.86 [n = 8] [*P*<0.05 versus saline-sham, EPA+DHA-sham and EPA+DHA-IR]; EPA+DHA-sham 34.32±0.6 [n = 6]; EPA+DHA-IR 35.12±0.44 [n = 6]) and IL-lβ (saline-sham, 70.08±2.44 [n = 4] pg/mL; saline-IR, 88.27±1.84 [n = 6] [*P*<0.05 versus saline-sham, EPA+DHA-sham and EPA+DHA-IR]; EPA+DHA-sham 81.02±0.66 [n = 5]; EPA+DHA-IR 80.82±1.66 [n = 6]) were drastically augmented in control rats subjected to IR and normalized by EPA plus DHA supplementation.

## Discussion

In agreement with previous reports, data presented in this study indicate that liver IR injury induced by 1 h of warm ischemia and up to 20 h of reperfusion is accompanied by an early (3 h) enhancement in liver NF-κB DNA binding, with up-regulation of the NF-κB signalling pathway and pro-inflammatory cytokine expression (20 h) [3], [11], [20], and concomitant enhanced serum levels of these NF-κB-dependent pro-inflammatory products [3]. This latter effect of IR is due to Kupffer cell activation with increased cytokine production and release, which in turn may lead to systemic inflammation, a major event in IR liver injury [21]. In line with these observations, suppression of TNF-α release from Kupffer cells and decreased liver leukocyte recruitment have been related to the protection against IR injury afforded by the combined administration of Kupffer cells inactivator gadolinium chloride (GdCl_3_) and α-tocopherol [22].

Abrogation of liver IR injury after EPA plus DHA supplementation is evidenced by the normalization of serum AST and ALT levels and liver histology, thus confirming our previous observations [3], and studies in a rat liver perfused model showing improvement of the hepatic microcirculation that prevents cell death on reperfusion [23] or in macrosteatotic mouse liver [24]. Prevention of IR liver injury in n-3 PUFA supplemented rats is associated with recovery of NF-κB DNA binding activity, lost at 18–20 h of reperfusion [3], [9], [11], and with suppression of IR-induced mRNA expression of liver TNF-α and IL-1β and enhanced serum levels of these pro-inflamatory cytokines, in relation to non-supplemented animals. These findings provide further evidence for the anti-inflammatory properties of n-3 PUFA and are consistent with studies showing successful alleviation of hepatic IR injury after oral supplementation with n-3 PUFA-rich oil in a model of hepatic warm IR in rats, with significant diminution in liver leukocyte infiltration and reduction in the serum concentrations of TNF-α and IL-6 [25]. In line with these reports, increased n-3 PUFA tissue status in a model of experimental hepatitis diminished inflammatory liver injury, a response that was associated with reduced plasma TNF-α levels and hepatic gene expression of pro-inflammatory cytokines [26]. Furthermore, DHA has been reported to inhibit the activation of the NF-κB system in human umbilical vein endothelial cells activated by cytokines [27]. In addition, studies using a model of kidney IR, demonstrated partial reduction of renal disfunction in DHA treated mice, with increased intracellular PPAR-α expression and concomitant blockage of the NF-κB-induced TNF-α overexpression [28]. These findings point to PPAR-α-mediated attenuation of renal IR injury via reduction of the NF-κB-induced inflammatory pathway, a mechanism also underlying apoptosis in a model of cultured kidney cells subjected to hypoxia-reoxygenation [28]. NF-κB exerts a significant transcriptional control on TNF-α and IL-1β expression in Kupffer cells, which are in turn intimately involved in the response of the liver to severe stresses such as prolonged ischemia [29]–[31]. In agreement with our previous reports [3], suppression of the IR-induced enhancement in the NF-κB DNA binding activity at 3 h of reperfusion, was observed in the n-3 PUFA-preconditioned group. This was observed concomitantly with increased mRNA expression of the PPAR-α-regulated enzymes CPT-I and Acox, thus evidencing PPAR-α activation upon n-3 PUFA-preconditioning. These effects were paralleled by a direct interaction between activated PPAR-α and NF-κBp65 as shown by the formation of PPAR-α/NF-κBp65 nuclear complexes, with parallel decreased IκB-α phosphorylation in the cytosol. Taken together, these data provide a molecular mechanism for n-3 PUFA-induced normalization of NF-κB DNA binding activity increased in the early phase of liver IR injury, which involves (i) nuclear NF-κBp65 sequestering through the generation of PPAR-α/NF-κBp65 complexes, and (ii) cytoplasmic NF-κB sequestering through enhanced IκB-α stability promoting its association with NF-κBp65/p50 [15]. In agreement with this proposal, oxidized EPA has been reported as a potent inhibitor of cytokine-induced activation of endothelial NF-κB, via PPAR-α-dependent pathway and cytoplasmic retention of NF-κB p50 and p65 subunits [32]. Furthermore, studies in human endothelial cells have shown that the anti-inflammatory effects of n-3 PUFA in this experimental model occur via PPAR-α-dependent mechanisms regulated by oxidized EPA [33]. In view of these observations, it seems likely that the anti-inflammatory response elicited by n-PUFA preconditioning against liver IR injury, could be ascribed to inactivation of NF-κB via activation of PPAR-α exerted by oxidized EPA and DHA, biomolecules which readily undergo oxidation due to their polyunsaturated structure [34]. Oxidation products of EPA and DHA include E-series and D-series of resolvins synthesized by the cyclooxygenase and 5-lipoxygenase (5-LOX) pathway, which exhibit anti-inflammatory effects compared to those derived from arachidonic acid [35]. Although no direct effects of EPA and DHA oxidized derivatives on the stability of cytosolic IκB-α have been reported, resolvin E1 has been shown to attenuate the pro-inflammatory action of NF-κB and leukotriene B_4_ through binding to the G-protein-coupled receptors chemokine-like receptor-1 (GPCR) and leukotriene B_4_ receptor [36]. In this respect, at least two GPCRs are involved in transducing resolvin E1 signals, namely, ChemR23 and BLT1. In relation to resolvin D1 signal transduction, a lipoxin A_4_ receptor, and an orphan, GPR32, specifically interact with resolvin D1, an effect that may lead to the significant reduction of the TNF-α-stimulated NF-κB response in HeLa cells overexpressing GPCRs triggered by this DHA oxidation product [37]. Furthermore, DHA is metabolized by 5-LOX to form protectins, being protectin 1 the most potent anti-inflammatory isomer [38]. The significant protective role of n-3 PUFAs and their oxidation products is supported by the protection afforded against liver injury induced by carbon tetrachloride *in vivo* or hydrogen peroxide *in vitro* through protectin D1 and 17*S*-hydroxy-DHA formation [7]. Alternatively, EPA and DHA may undergo oxygenation by cytochrome P450 NADPH-dependent epoxygenation pathway, with formation of several epoxyeicosaquatraenoic acid and epoxydocosapentaenoic acid isomers, respectively, which might contribute to the anti-inflammatory effects of n-3 PUFA [35], [39]. Suppression of iNOS gene expression as a result of the interaction of IL-1β-stimulated rat hepatocytes with EPA and DHA peroxidized products, has been recently proposed as an alternative anti-inflammatory mechanism triggered by n-3 PUFA [40], thus evaluation of iNOS expression in relation to n-3 PUFA-mediated PPAR-α activation in an *in vivo* model of liver IR might be relevant. In addition to the anti-inflammatory effects of n-3 PUFA, enhancement of the hepatocellular antioxidant potential may also play a role against IR liver injury, considering that the non-enzymatic peroxidation of EPA and DHA leads to formation of ciclopentenone-containing J-ring isoprostanes (J_3_-isoprostanes) [41]. J_3_-isoprostanes react with sulfhydryl groups in Keap1 complex responsible for the ubiquitination and further degradation of transcription factor Nrf2, leading to Nrf2 nuclear translocation and expression of several liver antioxidant enzymes, glutathione formation, and diminution in lipid peroxidation rate [41], [42].

It is important to point that the immunosuppressive effects of n-3 PUFAs or fish oil supplementation have also been attributed to the elevated production of IL-10, an immunosuppressive mediator with important hepatoprotective effects [43]. Recently, chronic DHA supplementation has been found to reduce hepatocellular damage in a rat model of cholestatic liver injury, an effect that was attributed to down-regulation of NF-κB and TGF-β/Smad activities probably via interference of ERK activation [44]. Thus, the hepatoprotective and anti-inflammatory effects of n-3 PUFAs seem to be multifactorial, although the molecular mechanisms responsible for n-3 PUFAs effects in hepatic inflammation are not fully understood.

In conclusion, liver preconditioning against IR injury by n-3 PUFA supplementation is mediated by PPAR-α antagonistic effect with NF-κB-controlled transcription of pro-inflammatory mediators, leading to the recovery of NF-κB signalling activity and re-establishment of inflammatory cytokine homeostasis. Concomitant suppression of IR-induced liver oxidative stress by n-3 PUFA supplementation [3] may involve activation of Nrf2 signaling by J_3_-isoprostanes derived from EPA and DHA, with up-regulation of antioxidant cellular components. The results of this study support n-3 PUFA dietary supplementation as a novel non-invasive preconditioning strategy to protect the liver and other organs against IR injury. In this context, n-3 PUFA have been progressively obtaining major consideration as potential anti-inflammatory agents that may improve the prognosis of several chronic inflammatory diseases.
